# The Accuracy of the Microsoft Kinect V2 Sensor for Human Gait Analysis. A Different Approach for Comparison with the Ground Truth

**DOI:** 10.3390/s20164405

**Published:** 2020-08-07

**Authors:** Diego Guffanti, Alberto Brunete, Miguel Hernando, Javier Rueda, Enrique Navarro Cabello

**Affiliations:** 1 Centre for Automation and Robotics (CAR UPM-CSIC), Universidad Politécnica de Madrid, 28012 Madrid, Spain; alberto.brunete@upm.es (A.B.); miguel.hernando@upm.es (M.H.); 2 Universidad Tecnológica Equinoccial (UTE), Santo Domingo 230208, Ecuador; 3 Department of Human Health and Performance, Faculty of Sports Sciences, Universidad Politécnica de Madrid, 28040 Madrid, Spain; javier.ruedao@alumnos.upm.es (J.R.); enrique.navarro@upm.es (E.N.C.)

**Keywords:** kinect sensor, gait analysis, dynamic time warping, latency

## Abstract

Several studies have examined the accuracy of the Kinect V2 sensor during gait analysis. Usually the data retrieved by the Kinect V2 sensor are compared with the ground truth of certified systems using a Euclidean comparison. Due to the Kinect V2 sensor latency, the application of a uniform temporal alignment is not adequate to compare the signals. On that basis, the purpose of this study was to explore the abilities of the dynamic time warping (DTW) algorithm to compensate for sensor latency (3 samples or 90 ms) and develop a proper accuracy estimation. During the experimental stage, six iterations were performed using the a dual Kinect V2 system. The walking tests were developed at a self-selected speed. The sensor accuracy for Euclidean matching was consistent with that reported in previous studies. After latency compensation, the sensor accuracy demonstrated considerably lower error rates for all joints. This demonstrated that the accuracy was underestimated due to the use of inappropriate comparison techniques. On the contrary, DTW is a potential method that compensates for the sensor latency, and works sufficiently in comparison with certified systems.

## 1. Introduction

Despite the fact the Kinect V2 sensor is no longer manufactured, Kinect V2 devices keep working. There are hundreds of users around the world, many of them using the sensor for research in the field of biomechanics. Microsoft has stopped supporting the adapter, which may affect the development of commercial applications such as motion gaming, but the generic depth detection solutions will prevail, and the Kinect V2 sensor will continue to be used for research purposes. The use of the Kinect V2 sensor in human gait analysis has increased in the last five years. In general, the Kinect V2 sensor has been extensively used for kinematic gait analysis [1] and the identification of normal and pathological gait [2]. Some important studies have analyzed and validated the application of the Kinect V2 sensor for kinematic gait analysis [3,4,5]. Despite that fact, there is something interesting in these studies. In order to validate the data obtained by the Kinect V2 sensor, the data from certified systems are resampled using the timestamps of the Kinect sensor. Afterwards, the differences are measured by using a vertical point-to-point comparison, as in [6,7,8]; all these studies refer to Kinect V2. However, this is not the most suitable way to perform this process due to the system’s performance and the variable processing speed of the Kinect V2 sensor.

Variations in system performance and processing speed produce non-symmetrical temporal variations in signals. This problem is named latency. Latency is caused by several factors, such as the software development kit (SDK) settings, image resolution, image format, exposure, and even the graphics processing unit [9]. It is common in most depth sensor types, regardless of framerate, the brand and model (Kinect V1/V2, Intel Realsense, Orbbec, ASUS), the technology used (structured light, time of flight), and the processing algorithm (proprietary or general algorithm such as Nuitrack SDK). This makes the latency compensation technique that will be proposed in this study directly exploitable by other technology based on depth sensors.

The latency in the Kinect V2 sensor cannot be controlled via SDK, in contrast to the Kinect V1, wherein latency can be controlled indirectly by setting a smoothing parameter. In the case of Kinect V1, a decrease in smoothing parameter value produces a lower latency but also a less smoothed skeleton data. The search for a balance between these parameters makes latency inevitable.

Several studies have measured Kinect sensor latency for skeleton tracking applications. In [10] the Kinect sensor latency was found to be about 70 ms. On the other hand, in [11], three samples of latency were measured. These studies are related to Kinect V1, as we have not found studies dedicated to Kinect V2 specifically. This latency produces a dynamic lag and consequently an incorrect relationship between the points when applying a point-to-point comparison with the ground truth.

Several algorithms have been used to realign these time series during validations of Kinect V2 sensor. The most common procedure is the uniform temporal alignment. Among them are the Euclidean distance minimization, weighted Euclidean distance minimization [8,12], and even the cross correlation [13,14,15]. All of them have been applied for validation of Kinect V1/V2 during human gait analysis. However, the residual time scale variability can still be significant, even after modeling a uniform scale. Since the effect of latency is not a uniform delay, a uniform temporal alignment technique cannot be used to compensate for it. In these cases, a non-uniform temporal scaling technique is required.

Dynamic time warping (DTW) is probably the most successful algorithm with which to compare a pair of signals and measure their similarity, even if they fluctuate in speed. DTW [16] is a nonlinear time normalization technique based on dynamic programming. DTW allows the comparison of sequences with tolerance of small misalignments, enabling an accurate comparison.

DTW has been widely proven and validated in gait recognition applications [17] and the identification of human body gestures using Kinect data [18]. DTW has even been used for the validation of musculoskeletal models [19]. Commonly, DTW has been applied as a comparison distance, not as a measure of accuracy. That is because DTW is not a metric, it is a measure. However, it is possible to use this warped distance as an error metric if several conditions are imposed [20]. In our case, these conditions are oriented to the use of a constrained dynamic time warping within a Sakoe–Chiba band [16] that compensates only for the sensor latency.

Since the global time scaling used in previous validations of Kinect V2 sensor is not adequate to solve this problem, the purpose of this study was to explore the abilities of the DTW algorithm to compensate for sensor latency and develop a proper accuracy estimation. The accuracy results obtained with DTW alignment were always compared with those obtained by Euclidean matching.

Additionally, when validation is done by using only one Kinect V2 sensor, commonly the range of view of the sensor is a limitation. It forces the test subjects to walk at short speeds, altering the normal walking pattern. For this reason, some studies prefer the use of treadmills. However, treadmill analysis is unrealistic and should not be applied because it modifies gait pattern [21]. On this basis, disregarding the use of a treadmill, any gait analysis that uses a depth sensor must apply a multi-camera setup or use a mobile robot platform that moves the camera close to the participant while walking. In addition to the fact that the use of a treadmill is unrealistic, the variables that cause latency in the signals, among them the joint occlusion, may be hidden. The occlusion points are precisely where the camera SDK deduces the position of the joint, causing a greater latency in the signal. When using a treadmill, this would not happen, and therefore, we could not generalize by saying that our results are applicable to gait analysis. In this context, because we wanted our results to be useful for future studies specifically using depth sensors, we decided to use a dual-camera setup to capture overground walking as realistically as possible. Thanks to this special setup, it is possible to capture longer tracking ranges without altering the normal walking pattern.

## 2. Methods

### 2.1. Data Collection

**Figure 1:**
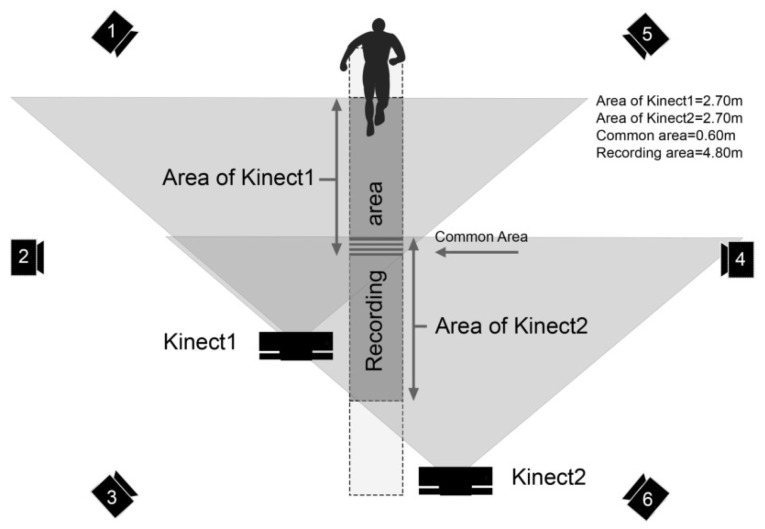
An overview of the workspace; the dual Kinect V2 system and the six Vicon cameras are shown. Each Kinect V2 sensor has a depth range for skeleton tracking of 2.70 meters. An overlapped field of view of 0.60 meters was reserved for the transition between the signals. The final walking path (recording area) had a length of 4.80 meters. More details about this configuration can be found in [25].

A dual Kinect V2 system was configured to retrieve the kinematic joint information of 25 joints at 30 fps. With the use of a single-camera system, the sensor’s range of view was not enough to capture at least one gait cycle with each foot. In addition, 6 samples on each side of the recording were unusable and disturbed. This was because during a very short period, less than the entire body was captured by the camera, which caused the algorithm to fail when building the skeleton. This effect was produced by the progressive apparition of the subject. For this reason, some studies such as [1,22] kept the subject on their feet within the recording area before starting the walking test, or alternatively, preferred to use treadmills [23,24]. In this study, a dual camera system was chosen to extend the range of view of the camera. The main goal of this configuration was to perform a validation of Kinect V2 sensor on a real walking in long workspaces. An overview of the workspace is shown in Figure 1. More details about this configuration can be found in our most recent study [25].

During the experimental stage, six iterations were performed using the Kinect system and a gold standard Vicon system. The Vicon system was composed of six M2 MCAM cameras with a sampling rate of 120 Hz. This was used as the reference measure.

### 2.2. Data Processing

**Figure 2:**
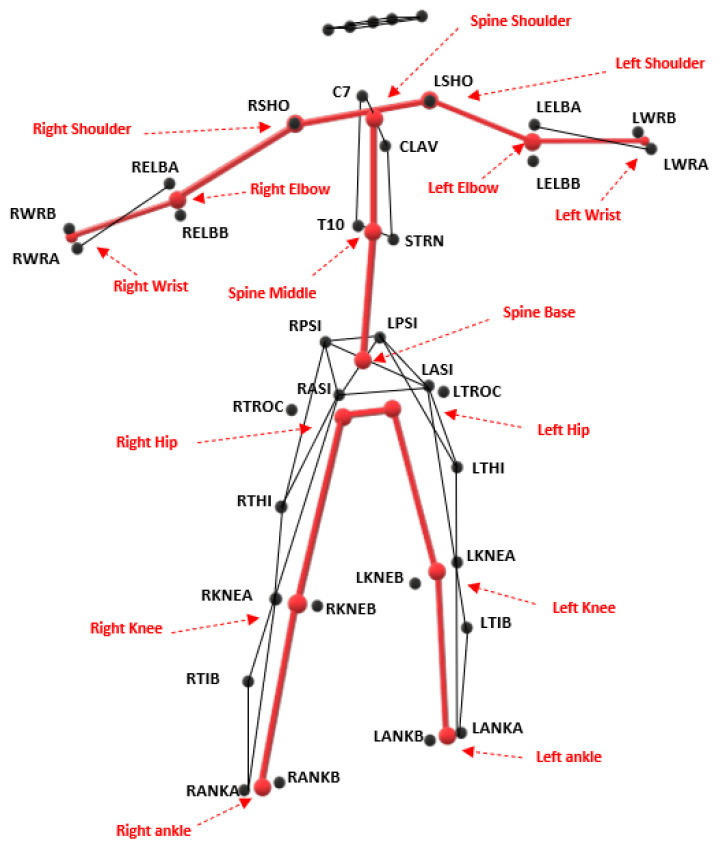
The Kinect V2 skeleton model built from Vicon markers.

We mapped the Vicon skeleton to build one equivalent to the Kinect skeleton. Mapping rules similar to those used in [26,27] were adopted, with the exception of the hip joint, for which the hip joint centering algorithm recommended by [28] was applied. The mapping rules are shown in Table 1. Figure 2 illustrates the equivalent skeletons. In this mapping process, six reflective markers were not considered (LTROC, LTHI, LTIB, RTROC, RTHI, and RTIB). The kinematic angles of the main joints involved in gait were analyzed in the sagittal, frontal, and transverse planes.

The time series from Kinect and Vicon systems were time-synchronized. That was done on the basis of the initial timestamp of the first gait cycle retrieved from the inter-ankle distance according to [29]. Afterwards, the Vicon data were subsampled from 120 to 30 Hz using a linear interpolation method and the timestamps of Kinect data.

Once the signals were pre-processed, the accuracy of the Kinect V2 sensor was analyzed by using Euclidean alignment and DTW alignment. To perform DTW alignment, an accumulated distance matrix *D* was built. It represents the costs of aligning the Kinect and Vicon time series. Each element in the accumulated matrix was built from the distance between corresponding points plus the minimum value of the previous neighburing elements. Given two time series, *k* and *v*, corresponding to one specific joint excursion each measured by the Kinect and Vicon systems respectively, the accumulated distance matrix *D* was built using the following formula [30]:

(1) $$D\left(i,j\right)=|k_{i}-v_{j}|+min\left\{\begin{array}{c} D(i-1,j)\\ D(i-1,j-1)\\ D(i,j-1) \end{array}\right.$$

where *i* and *j* represent the positions of the elements in the matrix *D*. Since the signals were previously synchronized and interpolated, the time series *k* and *v* have the same length and therefore D is a square matrix.

Once the matrix is built, the algorithm determines a warping path *W* over this matrix that allows aligning both signals to produce the minimum cost. DTW makes it possible to set a warping constraint *w*. In this case the DTW algorithm is called the constrained DTW and the value of *w* defines the limits of the Sakoe–Chiba band [16]. It represents the maximum amount the warping path is allowed to deviate from the diagonal. It is equivalent to the warping window width. A value of the warping constraint *w* of zero is used to retrieve the Euclidean distance error. The chosen value for *w* can greatly affect the accuracy [31]. This is the main reason to prefer metrics (such as the Euclidean distance) over measures (such as the warped distance). The research community is aware that DTW is a measure of distance rather than a metric, because DTW does not address the inequality of the triangle [31,32]. However, in the limit, as the warping constraint *w* approaches zero, DTW tends to be a metric [20]. For this reason, a very small value that compensates only for the sensor latency was set for the warping constraint *w*. This ensures the avoidance of unrealistic deformations in the signals that disturb the reported accuracy.

When the problem to be solved is unknown, the value of *w* is arbitrarily taken as a percentage of the signal length. In our case, the sensor latency could be measured using the data collected. In this context, the time shift between the corresponding peaks and corresponding valleys from the inter-ankle distance was measured. The inter-ankle distance was used for this purpose because it is a general measure for estimating the durations of gait cycles [29]. As result, the measured latency between the Kinect and the Vicon system was 78.41 ms with a standard deviation of ±48.82 ms. As can be noted, latency is not a uniform value. Latency changes between signals from different experiments and even within the same signal.

The setting of the w parameter was done experimentally. When w = 0 samples, then a point-to-point Euclidean comparison occurs. As the value of w increases, e.g., w = 3 samples, an elastic deformation of the signal is allowed in the time domain. If w is increased too much, the temporary deformation is not controlled and the algorithm may (not necessarily) erroneously match, e.g., a peak of flexion with the corresponding peak of flexion of the next gait cycle. However, this error would be evident when observing the warped signals during the analysis stage. According to a normal statistical distribution, for 68% of cases the latency varies between 29.58 and 127.22 ms, which could translate into a bandwidth varying from one to four samples. Ideally, the w parameter should be set to four samples, but it was decided to leave a margin of safety of one sample to ensure that there are no unrealistic temporary deformations. Therefore in this study the signal could be temporarily warped in a range of ±3 samples. If w had been set at four samples, the results reported in this document would have been slightly better, but we would have worked within an unrealistic deformation limit.

**Figure 3:**
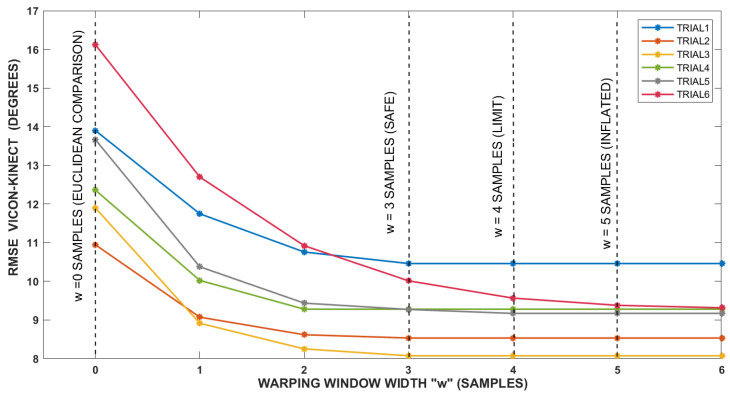
Setting of w parameter (the warping window width).

Figure 3 shows graphically the setting of w parameter. In this figure the warping window width increases from zero to six samples while the RMSE error corresponding to a specific joint excursion is analyzed (the knee flexion/extension). Figure 3 shows how the RMSE error decreases as the w value increases. The strongest decreases occur in the first four units of w; from there the curves practically flatten out, which means that the algorithm no longer has any effect. As mentioned above, a symmetric warping constraint *w* equal to three samples (equivalent to 90 ms at the sampling rate of Kinect sensor) was established. This value will compensate only for the sensor latency without disturbing the signals or the reported accuracy.

**Figure 4:**
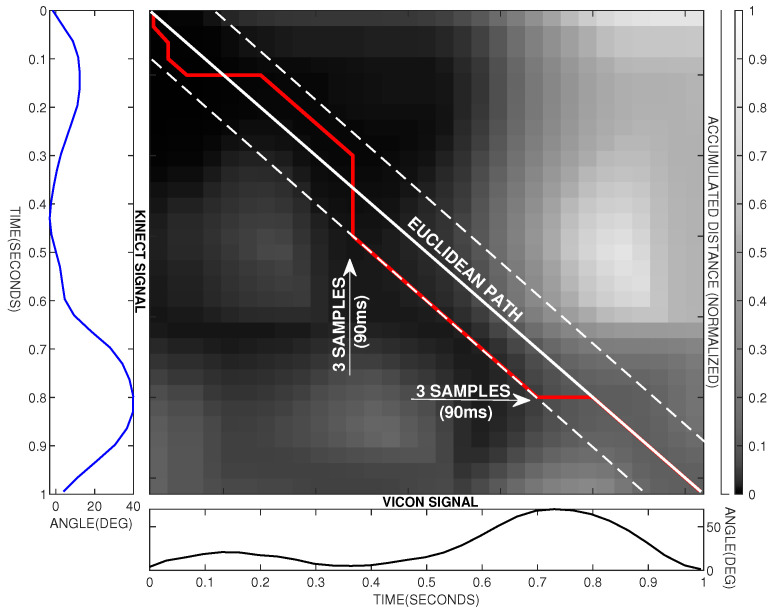
An example of how the DTW algorithm operates to find similarities between both signals. The movement belongs to the knee flexion/extension. The accumulated distance matrix obtained is shown in gray scale. The Vicon signal is shown as a black line and the Kinect signal as a blue line. The dotted white lines represent the limits of the Sakoe–Chiba band for a warping constraint w=3samples. The continuous white line represents the Euclidean path. The red line represents the warping path.

When applying a symmetrical warping window, a deformation at both sides from the diagonal of the Euclidean path (Figure 4) could be possible. This setting does not break the causality condition, in which the Kinect signal never could lead the Vicon signal. This is ensured because the point used for synchronization could be affected by a non-uniform delay that makes possible a misestimation of this point. Under this consideration, the Kinect signal can lead the Vicon signal and then the warping window can be set as a symmetrical window.

### 2.3. Data Analysis

Figure 4 shows an example of how the constrained warping path moves within the Sakoe–Chiba band when comparing the knee flexion/extension angle.

As can be seen in Figure 4, the DTW algorithm allows only three types of movement for the warping path *W* within Sakoe–Chiba band. These movements are horizontal, vertical, and diagonal. Considering a timestamp *t* in the time series *k* and *v*, if the movement is vertical, $v_{t}$ is extended to $v_{t+1}$ in the warped signal. If the movement is horizontal, $k_{t}$ is extended to $k_{t+1}$ in the warped signal. When the movement is diagonal, no temporary deformation is applied.

**Figure 5:**
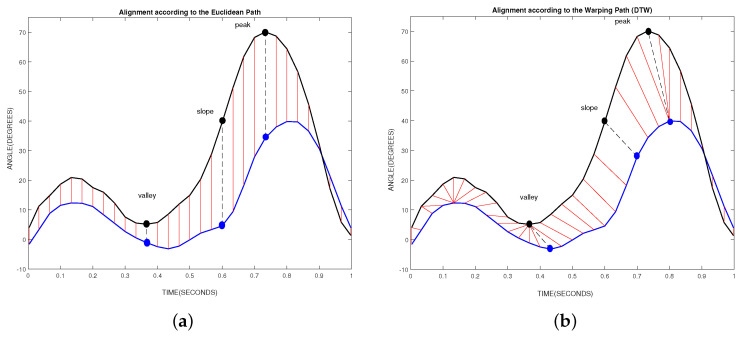
The alignment performed by the Euclidean and DTW algorithms. The movement belongs to the knee flexion/extension shown in Figure 4. The Vicon signal is shown in black line and the Kinect signal in blue line. The straight red lines indicate how the points are linked. There are three highlighted points: the peak, slope and valley from Vicon signal and the corresponding points in the Kinect signal. (**a**) The alignment of signals according to the Euclidean comparison. (**b**) The alignment of signals according to the DTW comparison.

Figure 5 shows the alignment of the signals performed by the Euclidean and DTW algorithms. The movement belongs to the knee flexion/extension shown in Figure 4. As can be seen in a point-to-point comparison in Figure 5a, the peak, valley and slope recorded by the Vicon system are compared with corresponding false points of the Kinect sensor. On the contrary, the correction showed in Figure 5b is evident. The DTW algorithm allows for the correct association of these points.

## 3. Results

**Figure 6:**
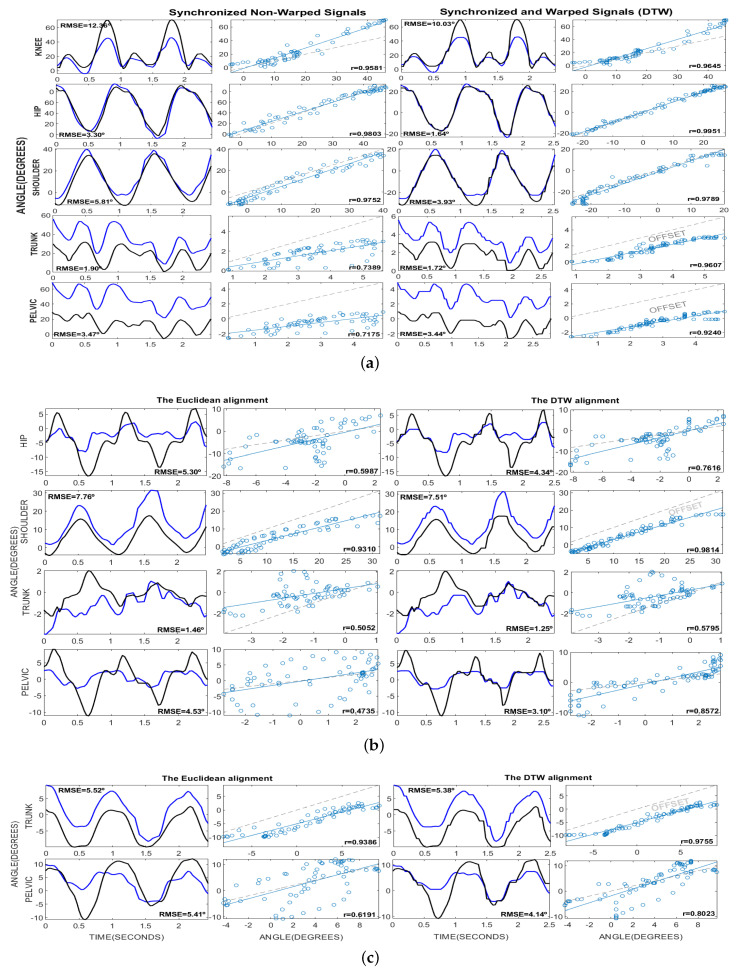
The kinematic angles for knee, hip, shoulder, trunk, and pelvis in (**a**) sagittal, (**b**) frontal, and (**c**) transverse planes reported from Vicon (black line) and Kinect (blue line) systems. There are four columns representing from left to right: The Euclidean alignment and the RMSE error. The scatter plot for this non-warped signals and Pearson correlation (r). The warped signals by using DTW alignment and the RMSE error. The scatter plot of this warped signals and the Pearson correlation (r). All these figures were made from one of the iterations gathered during the data collection. The warping constraint *w* was equivalent to 3 samples.

Figure 6 shows examples of joint excursions in sagittal, frontal, and transverse planes. All these figures were made from one of the iterations gathered during the data collection. In these figures it can be seen how DTW algorithm warps the time axis of the signals so that the corresponding features appear at the same location. As a result, the similarities between the signals are highlighted. In addition, it can be seen how the DTW algorithm is able to make a correction in the correlation of the signals. However, in some subfigures it is observed that an offset remains between the signals. This offset comes from the inevitable mismatch of the Vicon and Kinect skeleton models. This offset could be removed by applying a normalization by mean value, but we preserved this value in order to report the results as realistically as possible.

Table 2 summarizes the sensor accuracy estimated from a Euclidean comparison and a DTW comparison, respectively. The root mean square error (RMSE) but also the Pearson correlation (r) and a qualitative scale were used to describe results of each method. These values were averaged for all iterations performed during data collection. The average speed from all the iterations was 1.52±0.05 m/s. When both tables are compared, it can be noticed that results have lower RMSE values in the DTW alignment than in the Euclidean one. At the same time, the use of DTW allowed the signals to be better correlated in all cases.

In the sagittal plane, all joints had a ”HIGH” correlation when using DTW matching. Trunk and pelvis tilt changed their correlation from “MODERATE” to “HIGH” when DTW was applied. In general, all joints showed lower RMSE values. Results in both methods confirmed that the sensor works fine when retrieving excursions in sagittal plane. This is because the sagittal plane corresponds to the depth estimation by the sensor. As can be seen in Figure 6a, the flexion/extension of the knee, hip, and shoulder did not present significant offset with respect to the Vicon signal. On the contrary, the vertical displacement of the correlation line for trunk and pelvis tilt demonstrated a mismatch between the skeleton models. However, the value of this offset is shown in Figure 6a, and therefore could be removed from the values reported in Table 2.

In frontal plane, shoulder adduction/abduction showed the best correlation. Again, results in RMSE values were more promising for DTW matching. Nevertheless, the Figure 6b demonstrated the existence of a notable offset for shoulder adduction/abduction. As we explained above, this was produced by mismatches in the skeleton models of both systems. Although the correlation values for hip adduction/abduction, trunk sway, and pelvis obliquity increased significantly by using DTW, the values remained unpromising. The correlation in hip adduction/abduction and pelvis obliquity changed from “LOW” to “MODERATE”. The correlation in trunk sway remained “MODERATE”. However, the RMSE values stayed too high compared with the range of motion in these joints. This demonstrated that the sensor is unable to fully detect the amplitudes of movement for these joints. This is because, contrary to the sagittal plane, the estimated signals in the frontal plane correspond to the spatial estimation of the camera.

In the transverse plane showed in Figure 6c, trunk rotation demonstrated a ”HIGH” correlation despite the remarkable offset between the signals. On the contrary, the pelvic rotation continued with a ”MODERATE” correlation even after applying the DTW alignment. In addition, the pelvic rotation showed a notable RMSE error considering that no offset was observed in this signal.

### The Presence of Temporary Deformation Patterns

In addition to the remarkable improvements in system accuracy, the application of the DTW algorithm made it possible to differentiate the regions of the Kinect signal where temporary deformations were applied. To correct the Kinect sensor latency, DTW applies temporary deformations to the Vicon and Kinect signals. This would make its application impossible for future corrections wherein the Vicon signal is not present. However, the deformations applied on the Vicon signal can be transformed into opposite deformations on the Kinect signal. This means, for example, that a temporary contraction in the Vicon signal is equivalent to a temporary extension in the Kinect signal and vice versa. This allowed us to analyze the temporary deformation that should be applied only to the Kinect signal. At the same time, it allowed us to identify the presence of a pattern of temporary deformation using only the Kinect signal.

**Figure 7:**
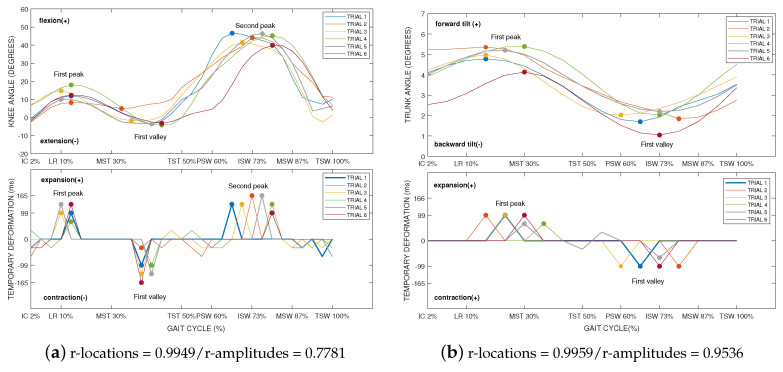
Examples of the kinematic joint angles and the corresponding temporary deformations represented only on the Kinect signal. The first Pearson correlation was calculated between the locations (r-location) of peaks and valleys of the signal and the locations of the corresponding temporary deformations. A second Pearson correlation was calculated between the amplitudes (r-amplitudes) of peaks and valleys of the signal and the amplitudes of the corresponding temporary deformations. To achieve a better understanding of the areas where temporary deformations occur, the phases of the gait cycle have been identified: initial contact (IC—2%), loading response (LR—10%), mid stance (MSt—30%), terminal stance (TSt—50%), pre-swing (PSw—60%), initial swing (ISw—73%), mid swing (MSw—87%), and terminal swing (TSw—100%).

The results of this analysis can be observed in Figure 7. For example, in Figure 7a, the gait cycle of knee flexion/extension signal is compared with its temporary deformation. The presence of temporary deformations in peaks and valleys can be noticed. An almost perfect Pearson correlation (*r* = 0.9949) indicates a strong relationship between the locations of peaks and valleys of knee flexion/extension and the temporary deformation. It can be noticed how the signal is temporarily extended during the peaks of flexion. Otherwise, there is a temporary contraction of the signal during the valleys. Therefore, we can generalize that the Kinect signal needs to be extended at the peaks and contracted in the valleys.

Furthermore, we analyzed the correlation between the amplitude of the signal at these points and the amplitude of the temporary deformation applied by the DTW algorithm. Results showed a high correlation (*r* = 0.7781) by which the magnitude of the temporary deformation is related to the magnitude of the signal at that point.

It was observed that this behavior was repeated in the remaining joints involved in gait. For example, in Figure 7b, the gait cycle of trunk tilt is compared with its temporary deformation. Again, the temporary deformations occurred in peaks and valleys. There was a high degree of correlation (*r* = 0.9959) between the locations of peaks and valleys and the temporary deformation. In the same way, the amplitude of the temporary deformation was related to the amplitude of the signal at that point (*r* = 0.9536).

To clarify the process to be followed in future studies, the correction of latency will have to be done during an offline analysis. To temporarily extend the signal during peaks, an interpolation process may be the most appropriate. A second order interpolation could be suitable given the nature of the signals we are processing. The amplitude of the signal to be reconstructed indicates the number of data that will be retrieved from the interpolation. Moreover, to execute a contraction in the valleys of the signal, surely the most appropriate process will be to design a median filter with a windowing equivalent to the amplitude of the signal at this point.

## 4. Discussion

The accuracy of the Kinect sensor has been a topic of interest in several studies. For example, in [22], Kharazi et al. presented a similar study to ours. The authors examined real overground walking. In this case, due to the position of the sensor (Kinect placed frontal to the person) the authors reported lower error rates than in our study. The authors reported an error of 6.31° for knee flexion/extension and 5.61° for hip flexion/extension (for overground walking at normal speed). However, placing the sensor in front of the person disturbs normal walking due to spatial limitations. Furthermore, the authors claimed that the two-meter walking path used in their study would be too short for future gait analysis applications.

In the study presented by Yunru et al. [5], during the joint kinematic validity, the author measured the error of the Kinect V2 sensor. Looking at the kinematic signals provided by the authors, it is remarkable, especially in knee flexion/extension, that the signals are affected by the sensor latency. This means that a vertical point-to-point comparison is not adequate for comparing the Kinect signals with the reference. In addition, the authors reported overly high errors due to the offset that existed between the skeletal models used. For this reason, we preferred to perform a comparative analysis with those results obtained by the authors after a calibration process. The authors reported 14.1±4.8° for knee flexion/extension, 11.5±4.1° for hip flexion/extension, and 4.7±2.2° hip adduction/abduction (for overground walking, at normal speed, with a single Kinect placed in front of the participant; the walkway being between 1.5 and 3.5 m from the Kinect). These values are very close to those we reported during the Euclidean matching (13.15° knee flexion/extension, 6.20° hip flexion/extension, 5.83° hip adduction/abduction). However, the differences are clear when we apply DTW (10.47° knee flexion/extension, 2.65° hip flexion/extension, 4.70° hip adduction/abduction).

In the study reported by Bilesan et al. [1], the accuracies of the most important kinematic variables from gait were reported. The correlation (*r*) and RMSE errors of the knee and hip flexion/extension, hip abduction/adduction, and pelvic rotation, were within acceptable ranges, which indicates the validity of the method used by the authors (inverse kinematics techniques). However, the correlation for pelvic obliquity and pelvic tilt was quite low. The signals were slightly noisy to ensure that the low correlations were due to the effects of sensor latency. The authors reported 1.699°/r=0.2679 for pelvic tilt, 2.288°/r=0.6641 for pelvic obliquity, 7.233°/r=0.9502 for pelvic rotation, 3.914°/r=0.9779 for hip flexion/extension, 3.240°/r=0.7562 for hip abduction/adduction, and 3.247°/r=0.9834 for knee flexion/extension. The data were collected with a single Kinect sensor and within a short walkway (overground walking, at moderate speed, with a single Kinect placed in front of the participant; the walkway was between 1 m and 3 m from the Kinect).

Finally, Bravo et al. [24], applied the DTW algorithm to compare the signals with the ground truth. Despite this fact, the signals from kinematic joint angles were too noisy. This produced errors rates higher than the reality. The authors reported 14.07° and 19.04° for hip and knee flexion/extension respectively, and 5.95° for hip abduction/adduction (at a self-selected speed and over a treadmill). They compared the Kinect and Vicon data similarly to our method, using a DTW algorithm. However, the authors did not mention anything about how they set up this algorithm. In addition, our method demonstrates better accuracy in capturing the kinematic joint angles.

As noted, the error rates that we retrieved when using a Euclidean alignment were very similar to those presented in previous studies. However, the improvement was noticeable after latency compensation using the DTW algorithm.

Additionally, this study revealed the existence of temporary deformation patterns in the signals to fit with the ground truth. These findings will facilitate the design of filters or machine learning algorithms to compensate for sensor latency in future studies. In addition, this study promotes the effective use of the Kinect sensor by proposing a way to improve its accuracy during human gait analysis.

## 5. Conclusions

The accuracy of Kinect sensor for human gait analysis was estimated using two methods of time series realignment. The Euclidean matching, which is the common procedure applied in earlier validations of Kinect sensor, was compared with the DTW matching. For this purpose, joint excursions in three planes were analyzed. Results from Euclidean matching were consistent with those reported in previous studies, whereas the DTW matching demonstrated considerably lower error rates. This demonstrated that the accuracy of the Kinect sensor has been underestimated due to the use of a Euclidean comparison.

DTW allowed the comparison of sequences with tolerance of small misalignments, enabling an accurate comparison. This demonstrated that DTW is a potential method that compensates for the Kinect sensor latency.

For future work, an in-depth analysis of the temporary deformation patterns in the Kinect signals could be conducted. These patterns could be applied in future studies to design filters or machine learning algorithms that compensate for sensor latency using only the Kinect data. In addition, this could promote the use of the Kinect sensor for human gait analysis in a safer way.

## Figures and Tables

**Table 1:** Mapping rules to build the equivalent Kinect V2 skeleton.

Kinect V2 Equivalent Joint	Vicon Markers
Spine base	Midpoint [RPSI,LPSI,RASI,LASI]
Spine middle	Midpoint [T10,STRN]
Spine shoulder	Midpoint [C7,CLAV]
Left shoulder	Marker LSHO
Left elbow	Midpoint [LELB marker A,LELB marker B]
Left wrist	Midpoint [LWR marker A,LWR marker B]
Right shoulder	Marker RSHO
Right elbow	Midpoint [RELB marker A,RELB marker B]
Right wrist	Midpoint [RWR marker A,RWR marker B]
Left hip	Hip joint centering algorithm [LASI,LPSI] ${}^{1}$
Left knee	Midpoint [LKNE marker A,LKNE marker B]
Left ankle	Midpoint [LANK marker A,LANK marker B]
Right hip	Hip joint centering algorithm [RASI,RPSI] ${}^{2}$
Right knee	Midpoint [RKNE marker A,RKNE marker B]
Right ankle	Midpoint [RANK marker A,RANK marker B]

${}^{1,2}$ The center of the hip joint’s location was calculated with the hip joint centering algorithm recommended by [28].

**Table 2:** The accuracy of the Kinect sensor for Euclidean matching and DTW matching. The RMSE and also the Pearson correlation (*r*) were averaged for all iterations performed during data collection. A qualitative scale with which to measure the degree of correlation present at each signal has been implemented. High: if *r* lies between ±0.80 and ±1. Moderate: if *r* lies between ±0.50 and ±0.79. Low: if *r* lies below ±0.49. The average speed from all the iterations was 1.52±0.05 m/s.

		Euclidean Matching			Dynamic Time Warping Matching		
		RMSE (°)	Pearson Correlation	Qualitative *r*	RMSE (°)	Pearson Correlation	Qualitative *r*
		(Mean ± SD)	(Mean ± SD)	(H./M./L.)	(Mean ± SD)	(Mean ± SD)	(H./M./L.)
**Sagittal**	Knee flex/ext.	13.15 ± 1.83	0.91 ± 0.04	HIGH	10.47 ± 1.49	0.94 ± 0.02	HIGH
	Hip flex/ext.	6.20 ± 2.08	0.94 ± 0.03	HIGH	2.65 ± 1.06	0.99 ± 0.01	HIGH
	Shoulder flex/ext.	9.53 ± 3.88	0.94 ± 0.02	HIGH	5.80 ± 3.39	0.97 ± 0.03	HIGH
	Trunk forw/back. tilt	2.09 ± 0.38	0.75 ± 0.02	MODERATE	1.93 ± 0.40	0.91 ± 0.04	HIGH
	Pelvis ant/post. tilt	3.22 ± 0.37	0.72 ± 0.07	MODERATE	3.14 ± 0.39	0.87 ± 0.06	HIGH
**Frontal**	Hip add/abd.	5.83 ± 0.99	0.41 ± 0.19	LOW	4.70 ± 0.94	0.63 ± 0.13	MODERATE
	Shoulder add./abd.	11.76 ± 3.98	0.87 ± 0.08	HIGH	11.69 ± 3.98	0.94 ± 0.05	HIGH
	Trunk right/left sway	1.31 ± 0.39	0.50 ± 0.19	MODERATE	1.10 ± 0.36	0.62 ± 0.18	MODERATE
	Pelvis up/down. obliquity	4.44 ± 0.48	0.40 ± 0.15	LOW	3.11 ± 0.42	0.76 ± 0.08	MODERATE
**Transverse**	Trunk int/ext. rotation	5.26 ± 0.64	0.88 ± 0.05	HIGH	5.09 ± 0.58	0.96 ± 0.02	HIGH
	Pelvis int/ext. rotation	5.88 ± 0.36	0.54 ± 0.14	MODERATE	4.81 ± 0.43	0.72 ± 0.12	MODERATE
